# Supplementary material for: An Implementation Science Framework to Develop a Clinical Decision Support Tool for Familial Hypercholesterolemia
Source: J Pers Med. 2020 Jul 23;10(3):67. doi: 10.3390/jpm10030067 (PMC7565418; doi:10.3390/jpm10030067)
Supplement: Supplementary file 1 [file jpm-10-00067-s001.pdf]

Supplementary Material

Supplementary Methods S1: Semi-structured interview guide used to conduct qualitative interviews with providers.

Introduction:

Good Morning/Good Afternoon Dr. /Mr. /Ms. __________. My name is [introductions of all study staff present]. Thank you for taking the time to meet with us today.

Warm-Up Chat (unstructured):

As we described in our email, our research team is currently working to develop a clinical decision support (CDS) tool for familial hypercholesterolemia (FH), which we plan to test next year in primary care practices in Rochester. This is your copy of the oral consent script that I would like to review with you.

Oral Consent Script:

You are being asked to participate in a research study which aims to engage stakeholders in the development of a Clinical Decision Support (CDS) Tool for Familial Hypercholesterolemia (FH). You are being asked to participate because you have been identified as a provider who may use CDS tools in the clinical setting. The main goal of this study is to obtain direct provider feedback on a prototype of a FH CDS tool.

If you agree to participate, we will ask you general questions about yourself and, and with your permission, record your responses on paper and through digital recordings. We anticipate this meeting will take approximately 60 minutes. The recording will be transcribed and only de-identified information will be shared. After transcription is complete the audio recording will be destroyed. For your time, you will be compensated with $100 (before taxes) and this will be added directly to your payroll deposit.

The risks of this research study are minimal, which means that we do not believe that they will be any different than what you would experience in your daily life.

The benefits which may reasonably be expected to result from this research study are a more efficient and user friendly CDS tool for use in clinical practice. Additionally, this project may assist providers in the diagnosis of patients with FH. Ultimately, patients and their family members who are found to have FH prior to a coronary heart disease (CHD) event will also benefit.

Please understand your participation is voluntary and you have the right to withdraw your consent or discontinue participation at any time without penalty. Specifically, your current or future medical care and employment at the Mayo Clinic will not be jeopardized if you choose not to participate.

If you have any questions about this study you can contact me at pencille.laurie@mayo.edu or Bangash.Hana@mayo.edu. If you have any concerns, complaints, or general questions about research or your rights as a participant, please contact the Mayo Institutional Review Board (IRB) to speak to someone independent of the research team at 507-266-4000 or toll free at 866-273-4681.

If a provider declines to participate, thank them for their time. If a provider consents, proceed.

A. Participant Demographics:

Okay, great. So, first we have a few demographic questions.

1. How long have you been in practice?
2. How many years here at Mayo?
3. Would you mind telling me your age?

B. Experience with CDS & FH:

Next, we have a few questions on your experience with CDS and FH:

1. Are you currently using electronic CDS tools to answer questions at the point of care (e.g. AskMayoExpert or MayoExpertAdvisor or UpToDate or any other resource)?
    a. (If yes) Which ones? What do you like or not like about them?
2. Do you think CDS tools save you time, provide value?
3. Do you feel the recommendations in AskMayoExpert are in line with your clinical practice/workflow?
    a. Could you explain further (why/why not)?
4. With your current understanding of FH, how confident do you feel that you would be able to detect a patient with a diagnosis of 'possible FH'?
    a. Prompts:
        i. Have you seen patients that you think may have had FH?
        ii. (If yes) Could you please describe why you suspected FH?
        iii. Did you search for information on FH?
        iv. (If yes) Where did you search for information?
        v. Did you order additional testing or refer the patient?
        vi. (If yes) To whom/where did you refer them?
5. Do you feel that it should be part of your work as a provider, to detect a patient who may have FH?
6. Do you feel that it should be part of your work as a provider, to manage an FH patient?
7. If you suspected an FH case, how likely would you be to order genetic testing to confirm the diagnosis?
8. Would you order the testing yourself?
    a. (If no) Who do you imagine would order the genetic testing?
9. How confident do you feel in your ability to successfully order the test?
10. Do you think a Genetic Counseling session should be involved in this scenario?
11. As a provider, how comfortable are you in discussing with a patient the possibility of a genetic disorder like FH?
12. Do you see the value of an FH clinic referral?
    a. Could you explain further (why or why not)?
13. What would some barriers be in you wanting to refer a patient to the FH clinic?
14. What could facilitate your referral to the FH Clinic?

Transition: Thank you.

C. Background Information on Familial Hypercholesterolemia (FH):

Before proceeding further, I would like to tell you a little bit about familial hypercholesterolemia (FH). Perhaps you already know this information I will be sharing, but we want to provide clinicians with the same background information.

FH is a genetic condition that is autosomal dominant and has a prevalence of 1:250 in the general population and a higher prevalence in certain populations such as French Canadians due to founder effect. It is a monogenic condition due to mutation in one of three main genes which include the *LDLR* gene, *ApoB* gene and *PCSK9* gene. FH predisposes to higher levels of cholesterol from a very early age and can subsequently lead to premature onset of cardiovascular disease such as myocardial infarction (20 times increased compared to general population). Currently there is low awareness about FH in patient and provider groups. It is estimated that up to 1.3 million people have FH in the US but less than 10% of them have been diagnosed. 90% remain undiagnosed. The Centers for Disease control and Prevention has labelled FH a Tier 1 genomic condition, meaning that case finding is warranted as it can lead to significant benefit. Being treatable with lipid lowering therapies such as statin, it is important to be able to diagnose FH early and initiate therapy. There is approximately 80% risk reduction with statin initiation. As part of the FH clinic, genetic testing and genetic counselor sessions help facilitate diagnosis and cascade screening the family members of a FH proband.

Transition: Do you have any questions before we proceed? Please feel free to ask for clarification now or at any point if any question seems confusing. We hope you feel open to express your thoughts and experiences honestly as we will use your comments to inform the design of the tool.

D. Case Scenario & Workflow:

As mentioned earlier, we have a very brief scenario for you to consider. A lipid profile was ordered for a 48 year old male patient. You received the results which showed an LDL-cholesterol of 200 mg/dL. This information and other criteria being met, the system will trigger you to receive a notification that this patient may have FH.

We would like to ask you some questions about your preferences in receiving the aforementioned information.

1. Could you briefly describe to us your usual workflow regarding a patient having a lipid profile test done?
2. In a scenario where the patient is not coming in for an appointment or has already been seen by you, now when would you want to receive the information regarding the patient being at risk of FH?
3. What would your next steps be?
4. In a scenario where the patient is coming for an in-person appointment, when would you want to receive information about the patient having a 'possible FH' diagnosis?
    a. Prompt:
        i. <u>If before visit</u>: When before the visit (day before; day of, prior to visit)?
    b. Could you explain why you prefer this?
    c. If you had questions about a patient's condition, would you prefer to search for information prior to their visit, during, after?

5. If you have a patient coming for a scheduled appointment, in which potential settings would you like/not like to receive an alert or in-basket regarding information about the patient having FH?
    a. Prompts:
        i. Follow up
        ii. Acute visit
        iii. Annual visit
        iv. Patient who is on your panel
        v. Patient who is not on your panel
    b. Could you explain to us a little bit about why you prefer this?
6. What format would you prefer to receive this information in?
    a. Prompts:
        i. An active alert, one that pop-ups during your usual workflow
        ii. A passive alert, one that can be viewed on chart review
        iii. An in-basket message
        iv. No alert or in-basket
        v. Other (e.g. combination, including a paper printout at time of rooming patient)
7. Do you think there is another member of the care team that might be able to assist you in conveying this information to the patient?
    a. Prompts:
        i. Help with family history?
        ii. Providing patient education information?
        iii. Could streamline the process for further referral?

Transition: I understand. This is excellent information and very helpful to us.

E. Alert Format:

<u>Based on your format preference for the alert</u>, we have a prototype here to show you and we would like to get your feedback on the prototype (proceed to show mockup of either the best practice alert or in-basket message—order in which each prototype will be displayed is based on provider preference indicated earlier in the interview). (Pause and allow provider to view the prototype)

Could you talk out loud and tell me what you are thinking or experiencing when viewing this screen?

Prompts to consider depending on providers' observations:

1. What do you think of the information provided in this alert?
2. What would you need to know and understand, to respond to the alert or in-basket?
    a. Does the alert have enough information for you to perform the necessary tasks?
    b. What are those tasks?
    c. Would you be comfortable with performing the tasks?
3. What information would you want added to the alert?
    a. Prompt:
        i. E-Consult?
4. What information would you want removed from this alert?

5. Would you want to have a snooze button on the message?
    a. If you snoozed, when should the message display to you again?
    b. If you snooze it, do you think this message should still show up for other providers?
    c. Could you explain why you think this?
6. In your normal workflow, how would you rule out the secondary causes of hypercholesterolemia?
    a. Prompts:
        i. If upon chart review, you find that the patient has a serum thyroid stimulating hormone (TSH) value (normal) but no tests or diagnosis codes indicating or ruling out liver disease or nephrotic syndrome, what would your next steps be as part of your usual workflow?
        ii. Could anything be added to the alert/message that could help you rule out secondary causes of hypercholesterolemia?
7. Is the AskMayoExpert knowledge resource enough for you? Any other resources?
8. After seeing the prototypes, do you think you would do anything differently in your workflow?
9. If you received an alert/in basket that your patient may have FH, would you want access to a patient decision aid?
10. Would you use the patient education materials?
11. Are you aware that Epic has a color coding scheme for their BPA's?
12. Having seen the information and the format, how would you prioritize this information based on the following schema? Red, at the top would be considered the most urgent and gray the least.

- ☐ Red
- ☐ Orange
- ☐ Yellow
- ☐ Pale Yellow
- ☐ Gray

Transition: Okay. This is good for us to know.

F. Post Interview:

1. We would like to hear your thoughts on what would help you to pay attention to this particular information or to use this information?
2. Would email or some communication from leadership be useful in encouraging you to use this information?
3. Would you want some kind of training to use the information?
4. What form of training would you prefer?

- ☐ Video
- ☐ Education Pamphlet
- ☐ Both
- ☐ None
- ☐ Other:

G. Closure: I understand. This has all been very helpful. I think that is all I need unless you have any other comments or questions. Thank you again for taking the time to provide us with your insights.

We will be using your feedback to develop and refine the FH CDS tool. Your remuneration will be deposited in your payroll check within 2-4 weeks. And if you think of anything else you would like to share with us, please reach out by email or by phone.

Supplementary Methods S2: Post interview implementation survey administered to providers to understand contextual variables that could influence adoption of the clinical decision support (CDS) tool into practice. Responses were documented using a 5 point Likert scale (completely disagree, disagree, neither agree nor disagree, agree, completely agree).

Implementation Survey (Post Interview)

Thank you for participating in the FH CDS Interview. We have a few additional questions to help us identify opportunities to enhance the FH CDS tool further. We think this survey will take about 2 minutes of your time.

Please indicate your level of agreement with the following statements concerning your experience with the FH prototype.

1. This tool meets my approval
2. This tool is appealing to me
3. I like this tool
4. I like welcome this tool
5. This tool seems fitting
6. This tool seems suitable
7. This tool seems applicable
8. This tool seems like a good match
9. This tool seems implementable
10. This tool seems possible
11. This tool seems doable
12. This tool seems easy to use
13. This tool will improve early diagnosis of patients with FH
14. I trust the quality and validity of evidence supporting this intervention
15. This tool meets my needs to provide needed resources to my patients
16. I recognize the importance of implementing this tool into practice
17. This tool is appropriate for ECH clinicians
18. This tool fits within my existing workflow
19. This tool will not increase the time needed with a patient
20. Implementing this tool is a good option to identify FH patients at Mayo
21. The implementation of this intervention within Mayo is important
22. This tool appears easy to access and incorporate into my workflow
23. This is a valuable tool for ECH clinicians
24. This tool will help me identify and refer or manage FH patients
25. It is important to me that cardiologists embedded in ECH continue to vet this tool

Please tell us any additional thoughts about your experience with the FH CDS tool

Survey Abbreviations: FH= Familial hypercholesterolemia, ECH= Employee and Community Health, CDS= Clinical decision support

Table S1: Comparison of implementation survey responses between primary care providers and specialist providers using Wilcoxon Rank Sum Test.

**Implementation Outcome Measures and CFIR Constructs Assessed**		**PCP (n=7) Score (Median, IQR)**	**Specialists (n=6) Score (Median, IQR)**	***P*-Value***
Acceptability of Intervention Measure (AIM)		17.0 (5.0)	17 (1.5)	1
Intervention Appropriateness Measure (IAM)		17 (1.5)	16 (0.75)	0.88
Feasibility of Intervention Measure (FIM)		16 (1.5)	16 (0.0)	0.83
Intervention Characteristics	Evidence Strength and Quality	4 (1.0)	4 (0.75)	0.67
	Relative Advantage	9 (2.5)	9 (2.0)	0.83
Outer Setting	Patient Needs and Resources	4 (1.5)	4 (0.75)	0.42
Inner Setting	Compatibility	13 (3.0)	12 (1.5)	0.65
	Relative Priority	10 (2.0)	9 (1.5)	0.86
	Access to Knowledge and Information	4 (1.5)	4 (0.0)	0.88
Characteristics of Individuals	Knowledge and Beliefs About Intervention	4 (1.0)	4 (1.5)	0.73
	Self-Efficacy	5 (1.0)	4 (0.75)	0.41
Process	Champions	4 (2.0)	3.5 (1.75)	0.88

Abbreviation: PCP, Primary care physician
